# A Concept of a Plug-In Simulator for Increasing the Effectiveness of Rescue Operators When Using Hydrostatically Driven Manipulators

**DOI:** 10.3390/s24041084

**Published:** 2024-02-07

**Authors:** Rafał Typiak

**Affiliations:** Faculty of Mechanical Engineering, Military University of Technology, 2 Gen. S. Kaliskiego Str., 00-908 Warsaw, Poland; rafal.typiak@wat.edu.pl

**Keywords:** teleoperation, simulators, sensors

## Abstract

The introduction of Unmanned Ground Vehicles (UGVs) into the field of rescue operations is an ongoing process. New tools, such as UGV platforms and dedicated manipulators, provide new opportunities but also come with a steep learning curve. The best way to familiarize operators with new solutions are hands-on courses but their deployment is limited, mostly due to high costs and limited equipment numbers. An alternative way is to use simulators, which from the software side, resemble video games. With the recent expansion of the video game engine industry, currently developed software becomes easier to produce and maintain. This paper tries to answer the question of whether it is possible to develop a highly accurate simulator of a rescue and IED manipulator using a commercially available game engine solution. Firstly, the paper describes different types of simulators for robots currently available. Next, it provides an in-depth description of a plug-in simulator concept. Afterward, an example of a hydrostatic manipulator arm and its virtual representation is described alongside validation and evaluation methodologies. Additionally, the paper provides a set of metrics for an example rescue scenario. Finally, the paper describes research conducted in order to validate the representation accuracy of the developed simulator.

## 1. Introduction

Unmanned Ground Vehicles (UGVs) are being more and more frequently used in demining and rescue scenarios. This is due to both the fact that these activities are often carried out in environments that are dangerous to humans, and the fact that the tools used in these cases are not adapted to be man-portable and man-operated [1,2,3,4]. This is a trend which will continue as we move closer and closer into machine-only autonomous rescue and demining operations. However, because of the complexity and the dynamic nature of such missions, we can still see a wide range of unmanned machines being deployed and operated remotely. This creates a need to train IED/EOD and rescue operators in order to increase the operational safety and effectiveness of unmanned units [5]. One of the methods to increase the number of possible training instances and reduce costs at the same time is the use of simulators [6,7]. Useful for training in a wide range of tasks, ranging from maintenance to manipulation, these solutions must use highly accurate virtual models in order to be effective [8,9]. The high level of model detail should cover both the robot’s or UGV’s base platform’s structure and its kinematics, and the functionality of its tools/manipulators, with a wide range of control options like speed and torque control [10,11,12].

As teleoperation is the most commonly used mode for controlling UGVs in demining and rescue operations, these types of simulators should not only provide sensor data commonly found in real-world products, like torque or vision sensors, i.e., RGB and RGB-D cameras, but also additional information which may be beneficial from a training standpoint [13]. Less common, but more relevant from that perspective, especially as computation capabilities increase, is the possibility of having deformable environmental objects and terrain [14]. Conducted analyses have shown that there are several simulators which offer extensive use case possibilities [15,16,17,18]. A solution commonly used in research is the MuJoCo [19]. A demonstrated use case was the ability to use a gripper-hand manipulation to solve a Rubik’s cube using a 24DOF robotic hand driven using tendons [20]. A different example could be the MuJoCo simulator which researchers have used to build and simulate robotic manipulators in order to check initial concepts [21] to then transition them into real-world systems [15,22,23,24]. The described simulator supports most of the functions required in mobile robots with the exception of inverse kinematics and path planning. For research related to the analysis of object collisions, Pybullet [25] is being widely used, especially in the dynamics of gripping [24], and for manipulation of deformable objects (e.g., fabric) [26]. Another possible solution would be Gazebo [25], which is mostly being used for research on manipulation robots [27,28]. While none of these studies rely on Gazebo to perform skillful manipulations, one study clearly extended the simulation to external algorithms to deal with non-rigid bodies. Gazebo provides a simulation environment with the necessary actuators and sensors to enable manipulation. It also provides the Robotic Operating System (ROS) support which provides forward and reverse kinematics packages as well as path and motion planning. Another possible solution is the CoppeliaSim. It is a robotics simulator with a range of user-centric features, including sensor and actuator models, as well as motion planning, and support for simple and inverse kinematics. PyRep was recently introduced as a Python toolkit for teaching robots. It has been used to manipulate, pick up, and place cubes with the Kinova robot arm [29]. While the above-mentioned simulators focus on manipulation tasks which are in the scope of this article, they mainly focus on the machine–environment interactions. It should be stated however, that it is not the only requirement for robot/UGV training simulators, and the focus should be placed mainly on the need to train operators with the Human Machine Interfaces (HMIs) they will inevitably have to use in order to carry out a rescue operation. Most of the previously mentioned solutions rely on consumer-grade controllers for operator input. While sometimes found in real-world solutions of certain robots, they lack the interaction levels a controller has with its control station, as it is being replaced by an interaction with an operation system of a PC.

The main aim of this work was to develop a simulator capable of accepting commands coming from a control station typically used when operating UGVs or robots for IED and rescue missions [2]. Additionally, emphasis has been placed on achieving a high level of accuracy when recreating a real-world robotic arm for IED missions. In order to do so, the robotic arm has been equipped with a sensor suite for special tracking. Alongside a proposed data mapping method, data recorded from those sensors have been used to recreate real-world behaviors of the robotic arm in the simulated environment and were also later used for accuracy validation of said model.

## 2. Materials and Methods

**Figure 1:**
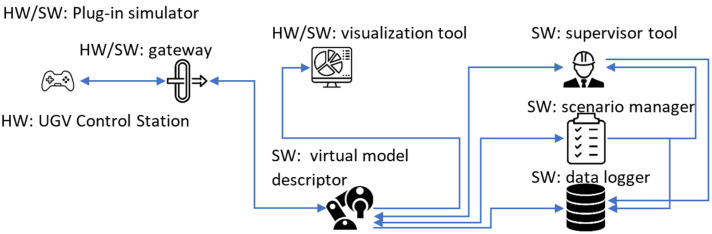
System structure of a plug-in simulator for rescue operations.

Studies have shown [30,31,32,33] that the increase of an operator’s immersion yields better learning results when using simulators. In order to take advantage of this fact, this paper proposes a solution which would allow for a plug-in simulator operation with the ability to assess mission critical parameters by an outside observer as well as to log test data for future analysis and the operator’s effectiveness tracking (Figure 1). The plug-in operation refers to the ability to connect an existing control station of a UGV to the simulator and to be able to send data between them just like it would happen during normal operation. Because of this, the system introduces a hardware/software component called a gateway. This device is meant to work bidirectionally and translate data between the communication protocol of the UGV and the simulator. To not disclose said protocols, the simulator offers an open communication protocol with the need for implementation of the gateway shifted to UGV manufacturers.

Within the simulator, data sent from the gateway is being processed by a software component called the virtual model descriptor. It is meant to house all the code used to model the simulated device and use it along with control signals to calculate its output state during simulation. That state is then sent to another software component called the scenario manager which houses all the virtual descriptions of objects taking part in the simulation. Its aim is to determine the ways in which the virtual model interacts with the environment on a scenario level. For example, if the scenario includes a person search and rescue operations, the object defined in the scenario manager may be a human model. Possible modifications to the model may include limb or body deformations, which the search and rescue operator should be able to detect prior to manipulations. A given set of parameters describing objects in the scene is then forwarded to another piece of software called the supervisor tool. The aim of this component is to provide the person supervising the training with sufficient data to enable performance assessment of the operator in real-time, and to help and guide him/her during testing, if needed. To do so, the supervisor tool is also able to access information from the virtual model descriptor. This allows to also measure the operator’s level of familiarity with the controlled device. The next component included in the plug-in simulator is the visualization tool. It is meant to be used by the trainee to control the virtual model in order to interact with the environment. The main requirement for this module is that it needs to represent the virtual device’s surroundings as accurately as possible. This covers both the hardware as well as the software side of things. The last piece of code running on the simulator is the data logger. Its aim is to record selected data for more in-depth analysis and post-mission assessment. That is why this component is receiving inputs from the virtual model descriptor, the scenario manager, and the supervisor tool.

### 2.1. Model Descriptor’s and Supervisor Tool’s Building Methodology

**Figure 2:**
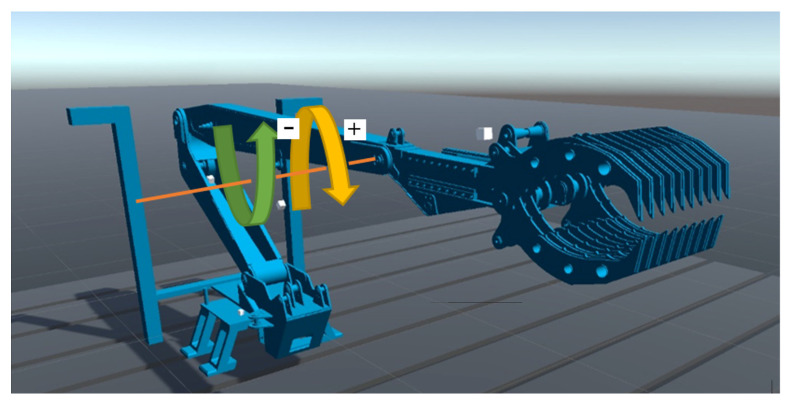
Testing angle directions used during the development of the DMD.

Testing methodology for the plug-in simulator consists of two main parts: the model’s building methodology and the experiment’s methodology. The first one describes the way the virtual model needs to be prepared to be able to be tested and the second one describes the way the model is going to be tested and how to process recorded data. The plug-in simulator assumes that the virtual representation and the real-world solution, which it is based upon, should resemble each other as closely as possible. This covers such aspects as kinematic description, manipulation functionalities, mechanical structure, physical dimensions, control scheme, and dynamic description. Because current robot development heavily relies on computer-based designs, developing all but the last two aspects is relatively easy. It is possible to export meshes of the robot’s components to external formats and reimport them into the game engine. Then, using built-in functionalities, stitch them together the same way it is being performed in real-life. The more work-intensive part is related to the last two aspects: control scheme and dynamic description. The first one requires a process of mapping the gateway protocol with the model, while the second one requires the development of a component called Dynamic Model Description (DMD). The creation of a DMD is a 3-stage process. The first stage is the parameter identification phase, carried out on the real-world unit. Its goal is to acquire time domain-based reference position data in the time domain from the controlled device based on a set of discrete control signals for each of the device’s functionality. An example of such an approach could be an IED/EOD manipulator arm (Figure 2). The data set should have twice the resolution of the control signal used to create the virtual model. The reason for it is that the unused datasets (every second datapoint in the dataset) will be used in the second (validation) stage. With the gathered data, an initial DMD can be developed ensuring that each joint can achieve the same movement speeds at the same positions in the time domain as its real-world counterpart. This creates a layered model for different control signal values. Its accuracy can be later increased by using approximation functions on each of the control signal speed and position datasets. For the purpose of this article, general polynomials have been assumed. By minimizing the approximation function using the sum of squared differences criterion, it is possible to obtain a continuous function of speed at certain rotational angles. To increase the DMD’s accuracy, it is possible to run this scenario in both directions, going from the starting angle to maximum and back for each of the joints (Figure 2).

**Figure 3:**
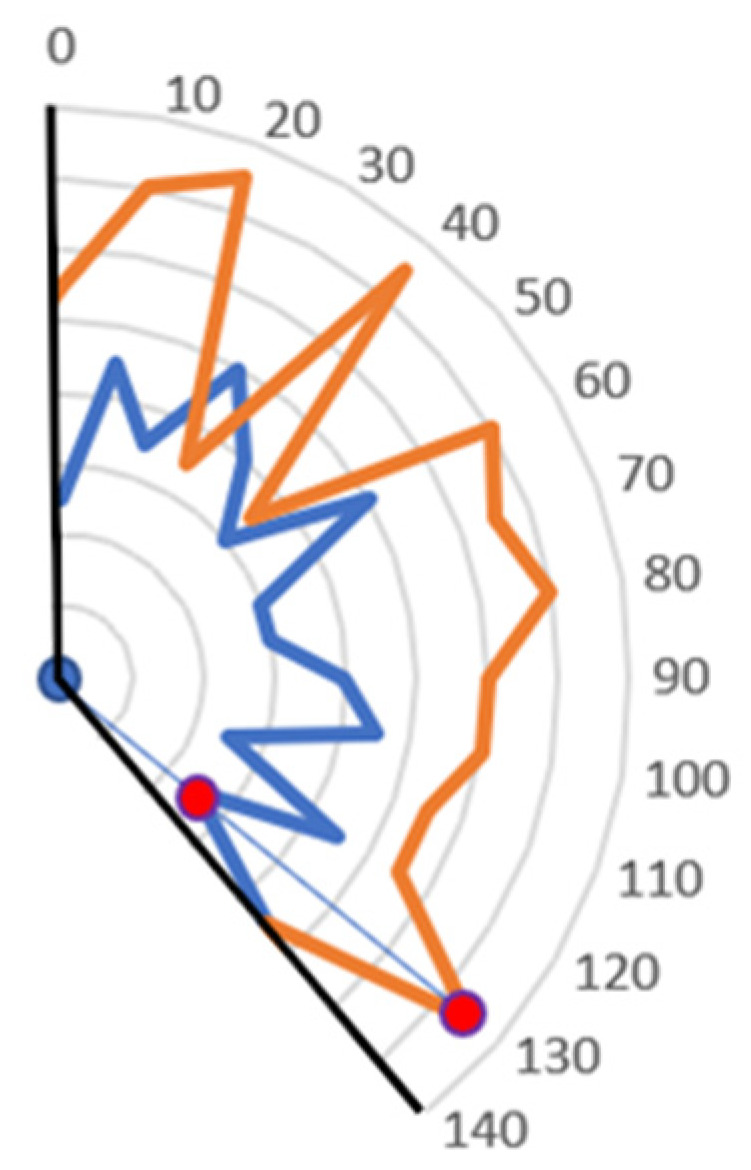
Visualization of the method for calculating angular joint speeds which are not covered by measured data. Colored lines represent speed values for consecutive, discrete control values. In order to calculate in between values, a line equation is being calculated based on two points from the known speed values, marked as red circles on the figure. The blue line symbolizes that line.

The above-described tasks should produce a layered DMD such as the one in Figure 3. While continuous for a set control signal, the DMD is still discrete in the control signal domain. In order to mitigate this problem, it has been assumed that a linear function would be calculated from two sets of known angular speeds for corresponding control signal values above and below the value currently generated by the operator. The output value is then proportional to the position of control signal value with relation to edge cases.

To generate data for the supervisor tool, the model needs to be configured in a way which enables data propagation between components. Table 1 lists the parameter types enabled for recording.

The same concept applies to the supervisor’s tool. Table 2 shows the data made available by this component. The list differentiates between a body and an object.

The testing phase covers not only the objective aspects of the rescue operation like precision of movement, equipment handling, and object handling, but also subjective ones. The reason for it is to measure the operator’s response to the training process and determine if he is becoming more familiar with the scenario. This knowledge is then used to determine two basic factors: environmental familiarity and scenario fatigue.

Transferring the operator’s control from a real-world solution to a simulated one introduces new stimuli for the operator. While a lot of care has been given toward a precise representation of the controlled object, the visual feedback the operator receives differs from what he will have to interact with. As such, there is a learning phase that needs to be carried out before the operator familiarizes himself with the simulator (environmental familiarity) and can start to learn behavioral responses which he will then be able to transfer over to the real world. The second aspect is scenario fatigue—it is a state where the operator knows all the specifics of a scenario and is becoming bored with it, which may introduce errors not present in real-world scenarios. In this case, a new scenario needs to be introduced to provide a new challenge and reintroduce uncertainty. For the above-mentioned purposes, two parameters were introduced that can be recorded: subjective accuracy and subjective situational awareness. The first one represents the operator’s assessment on how good he was doing during the test. The second one allows to determine how well the operator thought he knew about what was going on around the controlled object during the test. This assessment needs to be carried out after the operator has finished the test and should be recorded alongside the objective parameters.

### 2.2. Data Evaluation

Data evaluation is a process that is being conducted both in real time and after the tests. Its aim is to compute an overall effectiveness score for the tested operator. This includes objective, measurable factors as well as subjective aspects of an operator’s state and his or her perception of the completed task. The simulator uses a performance factor value to introduce a unified scoring system that has been described in detail in the author’s thesis [34] as a performance indicator (PI) methodology for teleoperated unmanned ground systems. The main requirement of this method is having a reference dataset. The original implementation has used manned operated units for that purpose. With the plug-in simulator, there is a referencing initial stage for each of the tested operators. It needs to be stated that not all of the previously listed object and manipulator parameters are being used in the PI. The remaining ones are either used for post-test assessments or for additional processing. Calculation of the PI is described using the following Expression (1):

(1) $${PI}_{x}=\frac{t_{x\_ref}}{t_{x\_y}}\left({no}_{col}+{nb}_{col}+{no}_{max}+{no}_{min}+s_{a}+s_{sa}\right)k$$

where:

${PI}_{x}$—performance indicator index;
$t_{x\_y}\;$—test duration;
$t_{x\_ref}$—reference test duration;
${no}_{col}$—number of object collisions when the force exceeded max value;
${nb}_{col}$—number of body collisions when the force exceeded max value;
${no}_{max}$—number of overloads in the max direction;
${no}_{min}$—number of overloads in the min direction;
$s_{a}$—subjective accuracy;
$s_{sa}$— subjective situational awareness;
k—completion indicator.

Test duration ($t_{x\_y}$) is the timespan calculated from t_start_ and t_stop_. Reference test duration ($t_{x\_ref}$) is calculated in a similar fashion but for the reference test phase. The completion indicator (k) is a 0 or 1 value given out per test by the supervisor via the supervisor tool. All the other components of the PI have certain weights attached to them. This was conducted in order to allow for higher testing flexibility if the operator is intended to focus more on a certain operational parameter. Setting these weight values is conducted freely, however, it needs to be recorded alongside the dataset. By default, the weight values per objective parameters equal 20 and per subjective 10. An example of this implementation is shown in (2).

(2) $${no}_{col}=20-5\sum_{y=1}^{n}j_{\mathrm{cx}\_y}$$

It has been assumed that, for the purpose of this simulator, the maximum number of mistakes an operator can make equals 4. If he exceeds that number, the calculated component’s value should not be calculated as being below 0, but instead, such state should result in an automatic test termination with a k factor equaling 0, unless the supervisor states otherwise.

### 2.3. Hydrostatic Arm Model

The plug-in simulator was created using the Unity game engine. The main reason for it was that it supports a robust physics engine, which is being used to calculate world interactions in real time [7]. Additionally, thanks to its target audience, it is relatively easy to develop HMIs for both the operator as well as the supervisor.

**Figure 4:**
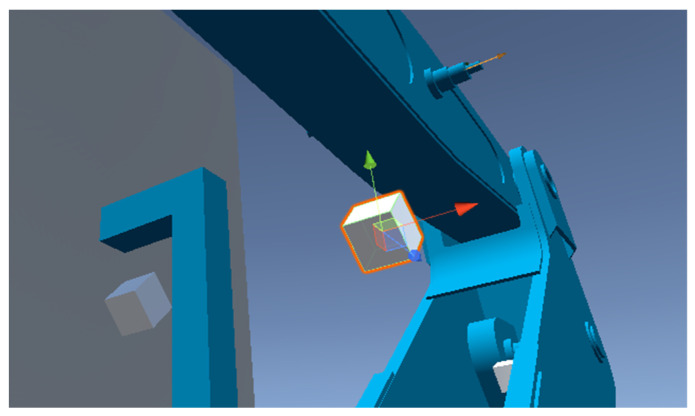
Hydraulic actuator’s representation in the virtual model with an anchor point on the arm.

In order to create a virtual representation of a hydrostatically driven manipulator arm, 3D construction models were used for mesh implementation. Next, a set of joints were created. They are a configurable physical constraint which forces a certain type of relation between connected objects. For the purpose of the manipulator’s definitions, joints were used both in a relation between the manipulator’s segments (i.e., an arm, boom, or a gripper) as well as between an actuator and a segment (Figure 4). Due to their configurable nature, it is possible to change their settings during simulation, which allows for the development of complex relations.

**Figure 5:**
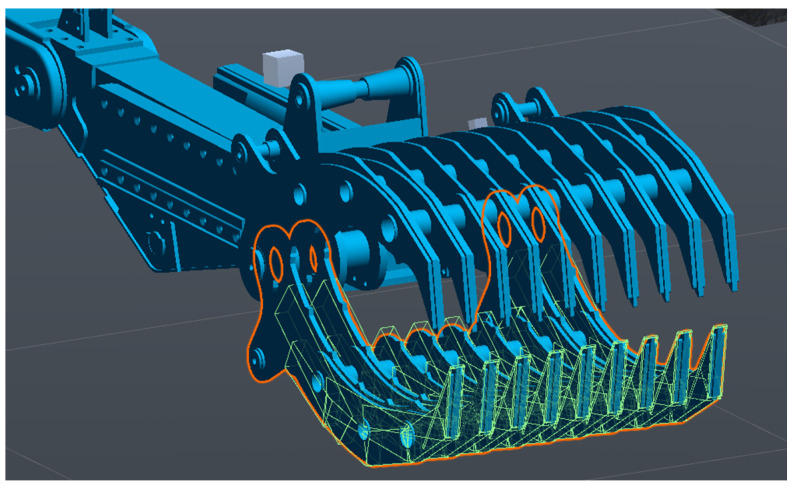
Structure of colliders on the manipulator’s lower jaw.

In order to enable world interactions, Unity uses constructs called colliders. They are a geometric representation used in physics calculations. It is separate from a mesh representation of an object because most of the time, the latter are too complex to allow for real-time computations. This does not mean that the manipulator’s model has rudimentary collision detections. Figure 5 shows the number of basic colliders being used for the lower jaw of the recreated hydrostatic IED/EOD manipulator.

**Figure 6:**
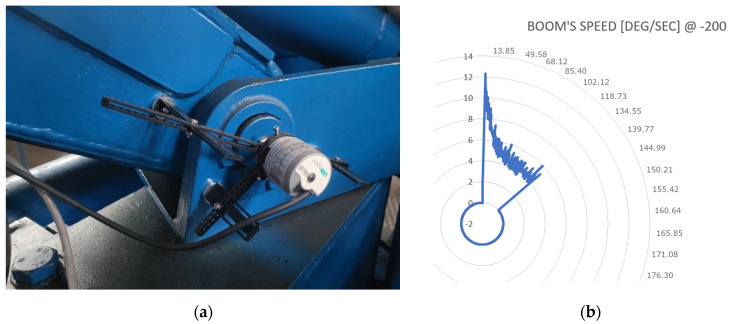
Boom’s speed identification: (**a**) Sensor setup and (**b**) Dataset for a control signal of “−200”.

The dataset for the simulator was gathered using absolute sensors (Figure 6a) connected to an input card. Gathered data (Figure 6b) were then processed to be used in the virtual model. Each manipulator segment’s speed was registered for varying control signal levels from −250 to 250, with a 10-unit step. This value span is compliant with the J1939 CAN standard, which is normally used with digitally driven hydraulic valves, like the PVED-CC series spool valves from Danfoss. The simulator was created using 40-unit step increments, with the same signal-level range. This means that real-world angular speeds of each of the manipulator’s segments were measured with valve control signals being: 0, 40, 80, 120, 160, 200, 250 and 0, −40, −80, −120, −160, −200, −250. In order to solve the problem of missing speed data for control signals in between consecutive control points (which correspond to the manipulator’s arm speed), the linear approximation method described earlier in this article was used.

### 2.4. Simulator’s Functionality

**Figure 7:**
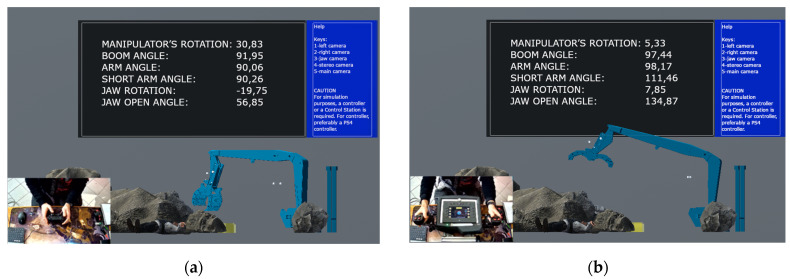
Simulator view of an operator using a standard controller (**a**) and a plug-in control station (**b**).

The main aspect of the developed simulator is its ability to interface with standard control stations. In Figure 7b, the solution presented is a CAN-bus-based UGV control panel, which is connected to the simulator PC using a specially developed CAN gateway. This solution acts as a translator between the proprietary control protocol and Windows API for HMI devices (used by the simulator).

**Figure 8:**
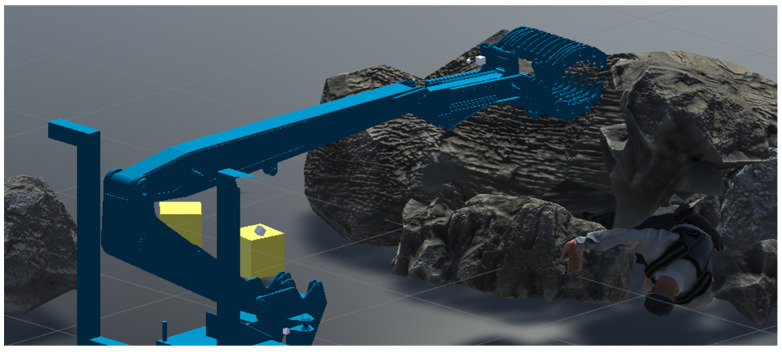
A rescue operation scenario created using the developed simulator.

The Unity-based simulator has the ability to utilize a wide range of objects to create complex scenarios. Figure 8 shows an example of a scene created for person retrieval tests.

**Figure 9:**
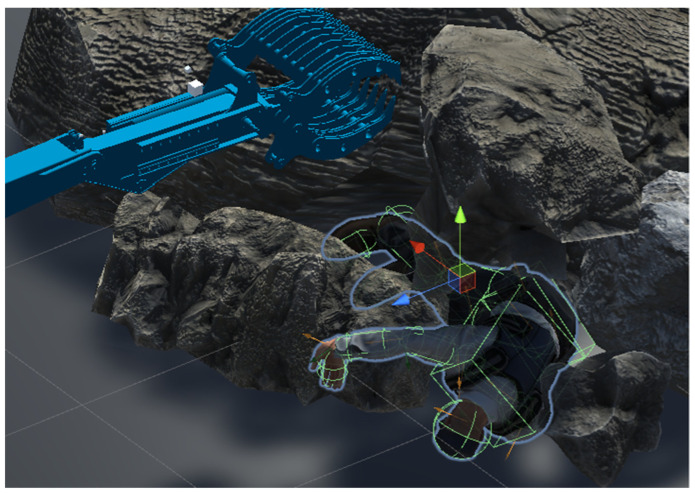
Collider structure of a male body model used for collisions during a rescue operation (green boxes and spheres).

As was the case with the manipulator, objects in the environment also possess physical traits, such as mass and dimensions. This is especially critical for interactions and assessing the operator’s effectiveness. While it is possible to use mesh colliders for environmental objects, using it on humans is not efficient due to how the skeletal structure is being managed in Unity. Because of this, the use of multiple simple colliders was required (Figure 9).

The simulator’s output is a timestamped file with all of the previously mentioned parameters logged for evaluation purposes. In order to automate the process of assessing and scoring the operators, a VBA script was developed which reads all the parameters, classifies them, and implements the evaluation methodology to produce an output Excel file with the final PI score as well as a score breakdown with regards to subjective and objective parameters. Additionally, a suggestion regarding the next aspect of the rescue scenario, where the operator needs to put more focus on, was generated.

## 3. Results

In order to determine the level of precision at which the virtual model represents the real-life solution, the following metrics were used: Mean Squared Error (MSE), Root Mean Squared Error (RMSE), and Mean Absolute Error (MAE).

The method of evaluating the developed simulator assumed additional real-world speed measurements were taken for each of the intermediate steps, with a constant increment of the control signal. This means that speed measurements for the following control signal groups were registered:

- 0, 20, 60, 100, 140, 180, 220.
- 0, −20, −60, −100, −140, −180, −220.

Afterward, each segment of the simulator was driven with the same control signal values and its angular speeds were measured. Later, both of these signal groups were compared using the previously mentioned metrics.

Table 3 presents the average values from each of the approximated levels for each of the manipulator’s segments. Of note is the fact that the highest levels of average differences in speeds are observed for the simulator’s boom segment and this trend persists for each consecutive segment. Several factors may be responsible for such an occurrence: approximation errors, simulator joint configuration, and weight differences between the model and the real-world unit. The last reason was considered the most probable because of the observed trend in MAE values. If it were the approximation errors, these values should not form a trend like that. It would be expected that they would remain somewhat constant between each segment. Joint configuration also does not provide a clear explanation as to why this trend is observed, but weight estimation errors could provide a reasonable explanation in that the simulation model stacks all the stack segments of a kinematic chain starting from the one being controlled. As such, if there would be a weight calculation error (said calculations were conducted using CAD designs and selected material information), that weight difference would affect each segment independently but would be summed when trying to move a stack of segments, rather than just one (i.e., jaws).

The maximum average residual variance levels of angular speeds of 0.0589 deg/s allow to determine that the simulator is not generating speeds which would produce unexpected results for the operator controlling it. It should be noted that the maximum speed errors obtained were for the Boom segment, with the highest control signal levels of 220 and equated to less than 0.1 deg/s (Table 4). This signal has produced an error of approx. 1.9% with the average Boom speed for that control signal being 5.048 deg/s.

The deviation levels, when compared to registered results, can be considered low, with a maximum value of 0.0244. This provides a stable foundation for a thesis, that the proposed method of transcribing real-world speed values onto a virtual model can be used without the model behaving “alien” or somehow “off” to the operator.

## 4. Conclusions

The plug-in simulator presented in this paper is a complete solution for the evaluating and training of rescue personnel in unmanned machine operation. By using existing control stations, it is possible for the operators to familiarize themselves with the HMI layout and control behaviors. The machine’s model implemented in the simulator strives to provide an accurate representation of its real-world counterpart through the use of functional identity, dynamic response levels, mechanical constraints, and environmental interactions. Additionally, an ever-growing repository of external objects allows for building complex scenarios with multiple factors and conditions being introduced. This is largely due to the usage of a commercially available Unity game engine. The research presented in this paper show that it is possible to create a fully functional plug-in simulator solution, which aims at faithfully representing the mechanical and dynamic aspects of the real-world unit.

## Figures and Tables

**Table 1:** Manipulator’s parameter list.

ID	Name	Parameter Name	Type	Visualized	Recorded
1	Joint angle	jax_y	Float	No	Yes
2	Joint speed	jsx_y	Float	No	Yes
3	Joint collision	jcx_y	Bool	Possible	Yes
4	Object name per joint collision	onx_y_z	String	No	Yes
5	Joint movement start	tjmx_y_start	Bool	No	Yes
6	Joint movement stop	tjmx_y_stop	Bool	No	Yes
7	Joint maximum position overload	tjox_y_max	Bool	No	Yes
8	Joint minimum position overload	tjox_y_min	Bool	No	Yes
9	Time start	tstart	Long	Yes	Yes
10	Time end	tstop	Long	Yes	Yes

**Table 2:** Environmental object parameter list.

ID	Name	Parameter Name	Type	Visualized	Recorded
1	Human body collision	h_cx_	Bool	Possible	Yes
2	Human body maximum force in collision point	h_cxf_	Float	No	Yes
3	Object collision	o_cx_	bool	No	Yes
4	Object maximum force in collision point	o_cxf_	Float	No	Yes
5	Maximum force on body without manipulator contact	h_cxf_nm_	Float	No	Yes
6	Maximum force on object without manipulator contact	o_cxf_nm_	Float	No	Yes

**Table 3:** Error table for each of the manipulator’s segments.

Manipulator Segment	MAE	MSE	RMSE
Boom	0.0589	0.0006	0.0244
Arm	0.0530	0.0004	0.0220
Long arm	0.0424	0.0003	0.0154
Short arm	0.0381	0.0003	0.0123
Jaws	0.0343	0.0002	0.0111

**Table 4:** MAE values per control signal value for the Boom segment of the simulator.

Control signal	20	60	100	140	180
MAE	0.0445	0.0424	0.0554	0.0665	0.0795
Control signal	−20	−60	−100	−140	−180
MAE	0.0401	0.0386	0.0406	0.0611	0.0694

## Data Availability

The data presented in this study are available on request from the author. Certain data may have share restrictions due to parts of the software being used in actions with restricted access.
